# Differences in Internalizing Problems Across Risk Groups of Smartphone Addiction: Simulation-Based Network Analysis of Intervention Targets

**DOI:** 10.3390/bs16081318

**Published:** 2026-08-03

**Authors:** Yi Huang, Wenwu Dai, Zhihui Yang

**Affiliations:** School of Humanities and Social Sciences, Beijing Forestry University, Beijing 100083, China

**Keywords:** internalizing problems, latent profile analysis, network analysis, smartphone addiction

## Abstract

Smartphone addiction (SA) has become a widespread issue and is consistently linked to a range of internalizing problems, including anxiety, depression, and sleep disorders. Previous studies have primarily focused on the overall association between SA and internalizing problems; however, differences in symptom patterns and potential intervention targets across SA risk groups remain unclear. This study recruited 1411 adults (*M*_age_ = 28.87 years, 45.64% female) who completed the survey. Data analysis was conducted using latent profile analysis and network analysis with simulation-based techniques. Latent profile analysis identified low-, moderate-, and high-SA groups. “Anxiety and panic” was the primary central node in low- and moderate-SA groups, whereas “Sleep disturbances” was the most central node in the high-SA group. Central bridge symptoms varied by SA severity: the “Depressed mood index” in the moderate- and high-SA groups and the “Somatic symptoms index” in the low-SA group. Simulation analyses identified specific anxiety and sleep nodes as candidate intervention targets, whose modeled changes were associated with variations in overall internalizing problems. Optimal targets differed by SA severity, generating hypotheses for future prospective research. These findings highlight the potential value of severity-sensitive, symptom-focused intervention strategies for preventing and alleviating internalizing problems across varying levels of SA.

## 1. Introduction

Advances in technology have positioned smartphones as a central medium for communication. Smartphones now support a wide range of activities, including commerce, social interaction, entertainment, and academic or professional tasks (Elhai et al., 2017; Tao et al., 2024). A recent report shows that China’s mobile internet user population has reached 1.121 billion, accounting for 99.6% of the total internet users (China Internet Network Information Center [CNNIC], 2026). Despite their convenience, smartphones are associated with a spectrum of detrimental behaviors, ranging from problematic smartphone use (PSU) to smartphone addiction (SA) (Y. Wang et al., 2025; Geng et al., 2021). Currently, SA has been conceptually framed as a behavioral addiction, mainly characterized by social dysfunction due to excessive use and other addiction-related psychological and behavioral impairments (Panova & Carbonell, 2018). Empirical research has documented its adverse effects on mental health and psychosocial functioning (Alotaibi et al., 2022; Cheng et al., 2024). A systematic review elucidated a strong association between SA and internalizing problems (Ratan et al., 2021). However, existing research has predominantly relied on variable-centered approaches, which may obscure individual heterogeneity (Alotaibi et al., 2022; M. Liu & Lu, 2022). To address this limitation, the current study adopts a person-centered approach to: (1) identify subgroups with distinct levels of SA risk; (2) examine the network structure and central nodes among internalizing problems (i.e., anxiety, depression, and sleep disorders) within each subgroup; and (3) identify potential candidate targets for tailored intervention strategies for each subgroup.

### 1.1. Relation Between Smartphone Addiction and Internalizing Problems

Prior studies have found that SA is closely related to internalizing problems (Karaş et al., 2023; M. Liu & Lu, 2022). Smartphone-induced stressors (i.e., privacy invasion, information overload, and information cocoons) are considered environmental stimuli that disrupt psychological balance; prolonged exposure to such stressors may tax attentional and emotional resources, increase vigilance, and impair recovery, thereby contributing to anxiety and depression (Lee et al., 2016). In addition, the social replacement hypothesis suggests that excessive smartphone use shifts social engagement online, reducing face-to-face interaction and offline social support, which may increase alienation and impair mental health status (Hall et al., 2018). Evidence has confirmed that social networking service addiction aggravates anxiety and depression by exacerbating adverse psychosocial conditions, such as increased loneliness, lower well-being and heightened social isolation (T. Wang et al., 2021). Given their portability and frequent usage around bedtime, smartphones significantly disrupt sleep routines. Proposed mechanisms include temporal sleep displacement via prolonged screen time, cognitive–emotional arousal from digital engagement, and circadian misalignment coupled with suppressed melatonin secretion because of exposure to blue light (Cain & Gradisar, 2010). A meta-analysis has found positive associations between SA, anxiety, depression, and sleep disorders (Li et al., 2020).

While accumulating evidence has suggested a positive correlation between SA and internalizing problems (Karaş et al., 2023), conventional variable-centered approaches may not sufficiently capture the heterogeneity and complexity within these relationships (Zeng et al., 2023). The implementation of person-centered analytical strategies, such as latent profile analysis (LPA), is thus methodologically crucial to distinguish SA subgroups and to elucidate their distinct associations with internalizing problems. Significantly, research has pointed out that LPA can enhance estimation precision in the absence of definitive clinical benchmarks (van Smeden et al., 2014). Recent studies using LPA have identified multiple at-risk profiles and examined their distinct associations with mental health outcomes. For example, Zeng et al. (2023) explored high-, low- and no-mobile phone addiction groups and there were significant distinctions in negative emotion scores among the three groups. Peng et al. (2023) classified problematic smartphone users into low-risk, middle-risk, and high-risk profiles, with the high-risk profile reporting the poorest mental health. Similarly, J. Wang et al. (2024) identified four internet addiction profiles, finding increased anxiety and depression in the most severe group compared to other profiles. The present study endeavors to explore latent groups of SA and to explore the discrepancies in internalizing problems across different groups.

### 1.2. Relation Between Anxiety, Depression and Sleep Disorders

Although research has indicated that the severity of internalizing problems varies across different SA or PSU groups through a person-centered perspective (Peng et al., 2023; Zeng et al., 2023), most studies rely on latent variable theory, assess disorder severity using total scores, and neglect the interrelations among the dimensions of internalizing problems (Fried & Nesse, 2015). The network analysis method (NAM) addresses limitations of conventional frameworks by conceptualizing disorders as networks of symptoms linked by dynamic relationships. It also enables the identification of core symptoms, whose amelioration may reduce overall symptom severity (Borsboom, 2017; Borsboom & Cramer, 2013). Accordingly, NAM is particularly useful for identifying critical symptoms, implementing precise interventions and improving treatment efficacy.

Anxiety disorders are distinguished by excessive fear and anxiety, whereas depression involves persistent negative affect and loss of interest, both leading to significant functional impairment (American Psychiatric Association, 2013). Anxiety and depression show a high degree of comorbidity and are dynamically interrelated; the occurrence of one disorder enhances the susceptibility of developing another (Garber & Weersing, 2010; Moffitt et al., 2007). Of particular concern are sleep disorders that frequently co-occur with anxiety and depression and constitute an important component of internalizing problems (Deng et al., 2021; Gardani et al., 2022). Previous studies have mainly focused on the overall association between SA and psychological issues (Luo et al., 2025); however, whether individuals with different profiles of SA exhibit distinct symptom patterns and require different intervention targets remains poorly understood. By integrating LPA with NAM, the present study is uniquely positioned to address this gap. LPA allows for the identification of subgroups with distinct patterns of SA, moving beyond arbitrary cutoffs on total scores. NAM then enables the estimation of symptom networks within each subgroup, revealing both the structural organization of internalizing symptoms and the identification of central and bridge symptoms that may serve as intervention targets. Furthermore, the simulation-based NodeIdentifyR Algorithm (NIRA) provides an approach to assess the potential impact of symptom-level interventions on the overall internalizing problems network (Lunansky et al., 2022). This combination of a person-centered method with simulation-based network analysis offers a novel framework for developing severity-sensitive, symptom-focused intervention strategies.

### 1.3. Current Study

While prior studies have utilized person-centered methods to investigate the association between SA and internalizing problems, these studies only conducted difference analyses, ignoring the structural network differences and interactions of internalizing problems’ dimensions across different SA groups. Furthermore, studies that use simulation-based network approaches to identify critical intervention targets for internalizing problems across different SA groups remain scarce. To address these limitations, the current study applies LPA to identify distinct SA profiles and implements NAM with NIRA to ascertain ideal targets for intervention. Compared to traditional linear methods, NAM elucidates the intricate dynamics among anxiety, depression, and sleep nodes, and NIRA simulates how alteration in specific nodes affects the entire internalizing problems network (Lunansky et al., 2022). This approach enhances clinical utility by highlighting key nodes and predicting system-wide effects of interventions (Machado et al., 2025).

## 2. Materials and Methods

### 2.1. Participants

The study adopted stratified random sampling to recruit adult residents (aged 18 and above) from all 16 districts of Beijing. Data were collected through both online surveys administered via Wenjuanxing (https://www.wjx.cn), a widely used online survey platform in China, and offline paper-based questionnaires. Detailed demographics are shown in Table 1. Of the 1450 questionnaires administered, a total of 1411 valid responses were received, resulting in a high response rate of 97.31% (45.64% female, *M*_age_ = 28.87, *SD*_age_ = 10.17).

### 2.2. Measures

#### 2.2.1. Smartphone Addiction Scale (SAS)

The SAS was used to assess SA severity (S. Su et al., 2014). It includes 22 items scored from 1 (“strongly disagree”) to 5 (“strongly agree”). Higher overall scores indicate greater levels of SA. The scale comprises six dimensions: withdrawal behavior (SA1), salience behavior (SA2), social comfort (SA3), negative effects (SA4), use of application (SA5), and application renewal (SA6) (e.g., “I would feel anxious if I could not use my smartphone for a period of time.”). The Cronbach’s α was 0.93. The reliability of this scale have been validated in the adult population in this study.

#### 2.2.2. Self-Rating Anxiety Scale (SRAS)

The 20-item SRAS was used to assess anxiety severity in adults (Zung, 1971). Five positively worded items (e.g., “I feel calm and sit still easily.”) are reverse-scored. The scale uses a 4-point Likert scale ranging from 1 (“little time”) to 4 (“most of the time”). This scale is divided into four parts: anxiety and panic (A1), vestibular sensations (A2), gastrointestinal/muscular sensations (A3) and somatic control (A4) (Olatunji et al., 2006). Higher scores imply more severe anxiety symptoms. The Cronbach’s α was 0.87.

#### 2.2.3. Self-Rating Depression Scale (SDS)

The SDS is a 20-item self-report questionnaire used to assess the severity of depressive symptoms in adults (Zung, 1965). Ten positively worded items (e.g., “My mind is as clear as it used to be.”) are reverse-scored. The scale uses a 4-point Likert scale ranging from 1 (“little time”) to 4 (“most of the time”). This scale is divided into four dimensions: well-being index (D1), depressed mood index (D2), somatic symptoms index (D3) and optimism index (D4) (Blumenthal, 1975). The Cronbach’s α was 0.81. For the well-being and optimism indices, higher scores imply lower levels of well-being and optimism. For the depressed mood index (D2) and somatic symptoms index (D3), higher scores indicate more severe depressive symptoms.

#### 2.2.4. Pittsburgh Sleep Quality Index (PSQI)

The 19-item PSQI was used to measure subjective sleep quality in the preceding month (Buysse et al., 1989; X. C. Liu et al., 1996). The scale comprises seven dimensions: subjective sleep quality (S1), sleep latency (S2), sleep disturbances (S3), use of sleep medication (S4), daytime dysfunction (S5), sleep duration (S6), and sleep efficiency (S7). Each dimension is scored from 0 (“no difficulty”) to 3 (“severe difficulty”). Higher scores imply poorer sleep quality. The Cronbach’s α was 0.74 in this study.

### 2.3. Data Analysis

#### 2.3.1. Latent Profile Analysis

LPA was conducted using Mplus8.0 to estimate one-class through five-class models to identify risk profiles of SA. Missing data were addressed using full information maximum likelihood. Model selection was based on three criteria: (1) Lower values of the Akaike information criterion (AIC), Bayesian information criterion (BIC), and adjusted Bayesian information criterion (aBIC) suggested better model fit. (2) Entropy values closer to 1 reflected greater classification accuracy, and (3) Significant *p* values for the bootstrap likelihood ratio test (BLRT) and the Lo–Mendell–Rubin likelihood ratio (LMR) test.

#### 2.3.2. Network Analysis

All statistical analyses were performed using R version 4.4.3. Symptom networks were estimated using the graphical least absolute shrinkage and selection operator (LASSO) combined with extended Bayesian information criterion (EBIC) model selection and a tuning parameter of γ = 0.5 (Epskamp & Fried, 2018). Networks were estimated using the EBICglasso method, which applies regularization to partial correlation matrices. To assess the comparative significance of each symptom across the network, node centrality indices were computed. Compared to strength centrality, expected influence (EI) more accurately reflects the influence of nodes in networks that include both negative and positive edges (Robinaugh et al., 2016). One-step bridge expected influence (BEI) was conducted to identify bridge symptoms that reflect connections between different disorder clusters (Jones et al., 2021). Network robustness was further analyzed using bootnet package in R (Epskamp et al., 2018). The correlation stability-coefficient (CS-C) was employed to evaluate the stability of centrality. As recommended, CS-C values above 0.25 were considered to demonstrate acceptable stability (Epskamp & Fried, 2018). The network comparison test (NCT) was employed to assess global strength and network structural invariance across three subgroups (Burger et al., 2023; van Borkulo et al., 2023).

#### 2.3.3. Simulated Interventions

An Ising model network was constructed to examine how interventions targeting each node influence the overall internalizing problems and to identify potential intervention targets. Following the approach of Jiménez et al. (2023), the data were binarized using the mean as the cutoff to represent favorable or adverse states of each node. Values below the mean were encoded as 0, indicating a more favorable state of the node, whereas values above the mean were encoded as 1, signifying a more severe state. The Ising model was then fitted, given its suitability for modeling changes in clinical characteristics. Subsequently, the NIRA was applied to generate 5000 simulated participants, and two types of intervention simulations were conducted for each node in the Ising network. The overall impact of each symptom-specific intervention on the internalizing problems network was evaluated using the total score index. Compared with the pre-intervention condition, the symptom associated with the largest absolute change in total score was considered to have the greatest potential preventive or therapeutic effect.

## 3. Results

### 3.1. Smartphone Addiction Categories

**Figure 1:**
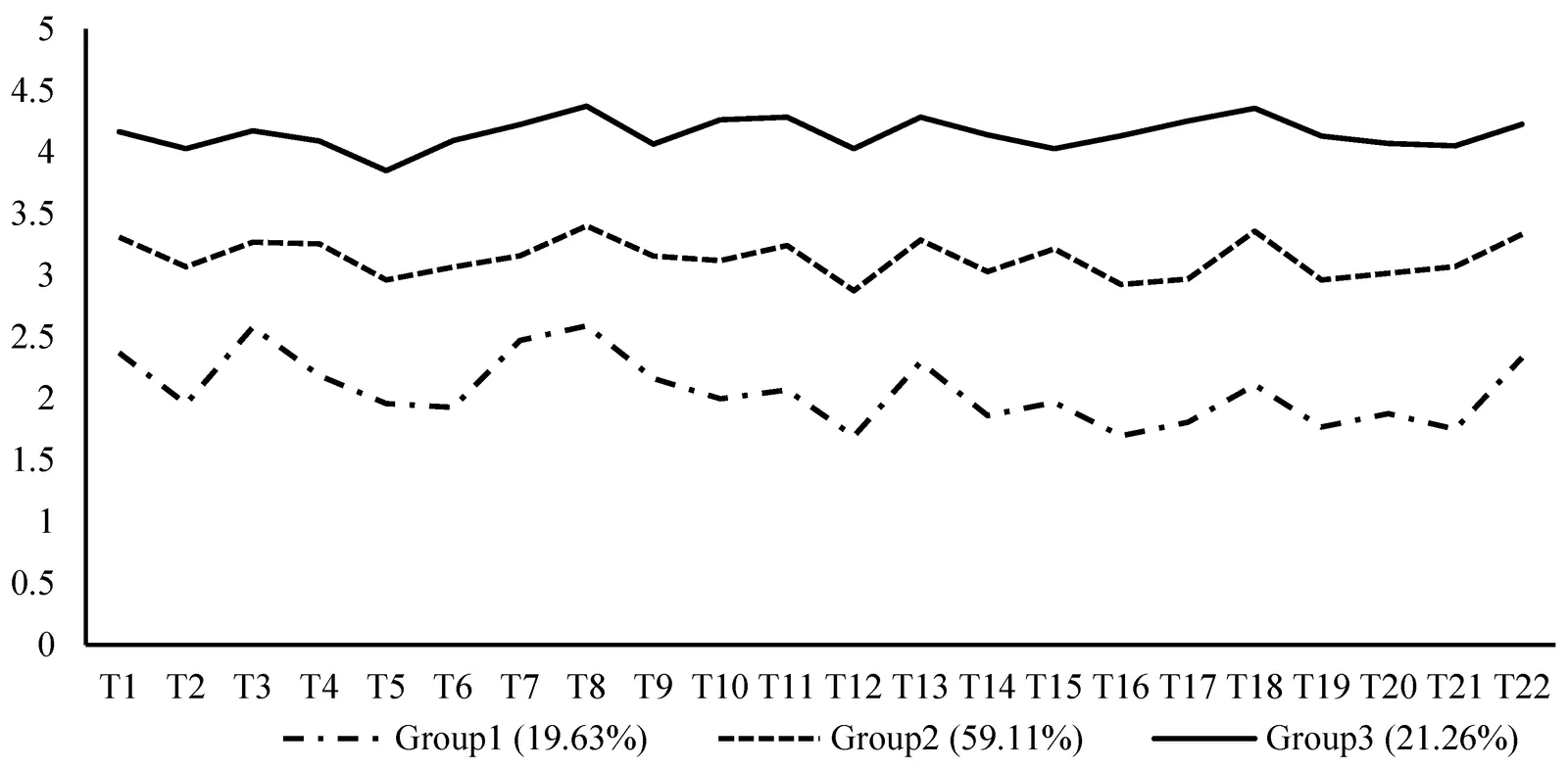
The three groups of SA by LPA. T1–T22 represent 22 items of SA inventory.

The three-category model had the highest entropy and lower aBIC, AIC, and BIC values, implying greater classification precision. Although the four-category model demonstrated lower aBIC, AIC, and BIC values, the largest decrease in information criteria occurred from the two-category to the three-category model, and the LMR tests for the four-category and five-category solutions were not significant (Table 2). Thereby, the three-category model was the optimum model. The average latent class probabilities for the three-class model were presented in Table 3. All participants had a mean posterior probability exceeding 0.90 for their assigned class, whereas the mean probabilities of membership in the alternative classes were below 0.10. These results further support the adequacy of the three-class solution. According to item-level patterns across the three profiles (Figure 1), the first profile (*n* = 277, 19.63%) showed the lowest SA scores across all items, indicating low-SA risk. The second profile (*n* = 834, 59.11%) showed moderate-SA levels, indicating moderate-SA risk. The third profile (*n* = 300, 21.26%) showed the highest SA levels, indicating high-SA risk. Accordingly, the three profiles were labeled as the low-, moderate-, and high-smartphone addiction groups.

The *M* and *SD* for each node across three groups are shown in Table 4. Significant between-group differences were observed in overall scores of SA, anxiety, depression, and sleep disorders (*ps* < 0.001). Post hoc comparisons verified that the patterns observed in each group were consistent with their defined features. A notable association was observed between group and gender (*χ*^2^ = 18.82, *p* < 0.001).

### 3.2. Network Structure Estimation

**Figure 2:**
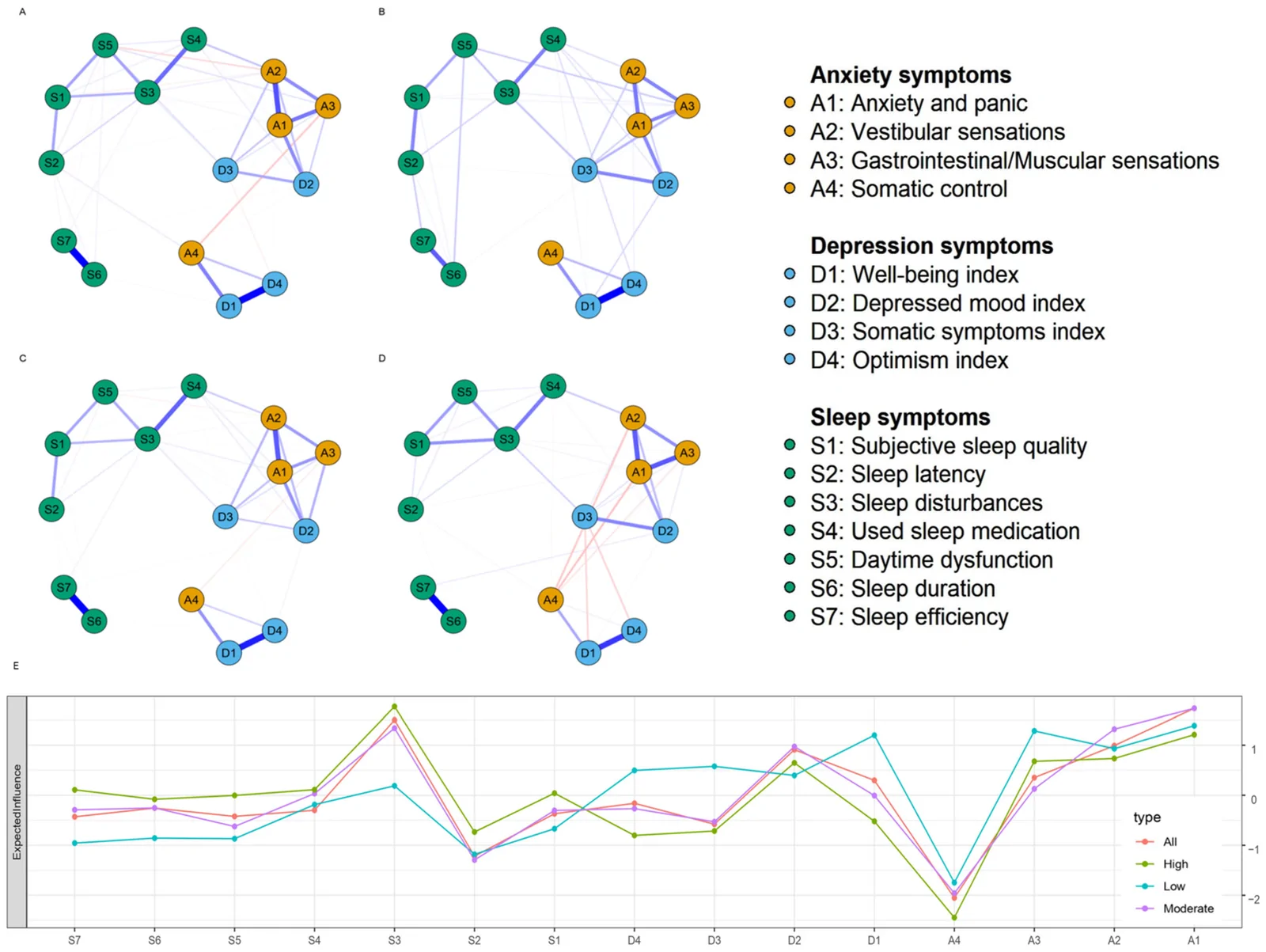
The anxiety–depression–sleep networks. (**A**) All participants; (**B**) the low-SA group; (**C**) the moderate-SA group; (**D**) the high-SA group; (**E**) the centrality of node EI; S: sleep quality; D: depression; A: anxiety.

The internalizing problems networks for all participants and the low-, moderate-, and high-SA groups are presented in Figure 2A–D. Several observations deserve mention. Of the 105 possible connections, the total sample network contained 78 non-zero edges, compared with 45 for the high-SA group, 66 for the moderate-SA group, and 55 for the low-SA group.

In the total sample as well as the moderate- and high-SA groups, the strongest edge was observed between S6 and S7 (“Sleep duration”—“Sleep efficiency”). In contrast, the strongest connection was observed between D1 and D4 (“Well-being index”—“Optimism index”) in the low-SA group.

The EIs for each node in all participants and three SA groups are presented in Figure 2E. In the total sample, “Anxiety and panic” (A1) became the most critical node (EI = 1.73), with “Sleep disturbances” (S3; EI = 1.50) following. In the low-SA group, “Anxiety and panic” (A1) also showed the highest centrality (EI = 1.39), followed by “Gastrointestinal/Muscular sensations” (A3; EI = 1.29). In the moderate-SA group, “Anxiety and panic” (A1) remained the core node (EI = 1.73), with “Sleep disturbances” (S3; EI = 1.34) ranking second. In the high-SA group, “Sleep disturbances” (S3) became the most significant node (EI = 1.77), succeeded by “Anxiety and panic” (A1; EI = 1.21).

**Figure 3:**
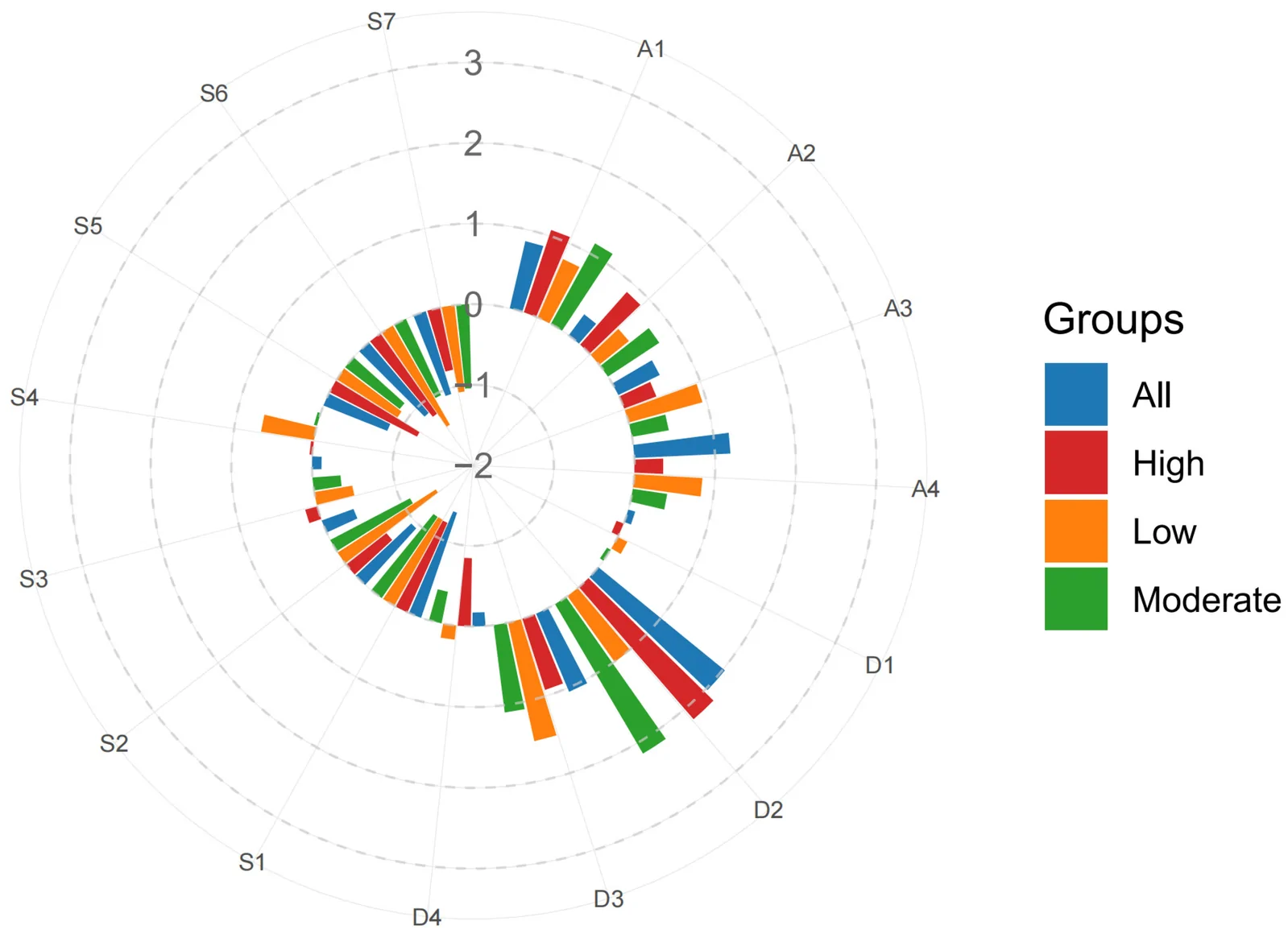
The bridge expected influence in internalizing problems networks among all participants and three subgroups.

BEI values for the total sample and three subgroups are presented in Figure 3. Bridge symptoms were identified based on a standardized BEI threshold of ≥1, which represents a commonly used convention (Sánchez Hernández et al., 2021). To allow for a comprehensive evaluation of the bridge symptom distribution, all BEI values are reported in full in Table S1. In the total sample, the most influential bridge symptoms were “Depressed mood index” (D2; BEI = 2.03) and “Somatic control” (A4; BEI = 1.19), followed by “Somatic symptoms index” (D3; BEI = 1.05). In the low-SA group, the strongest bridge symptoms were “Somatic symptoms index” (D3; BEI = 1.50) and “Depressed mood index” (D2; BEI = 1.00). In the moderate-SA group, bridge symptoms included “Depressed mood index” (D2; BEI = 2.15), “Anxiety and panic” (A1; BEI = 1.14), and “Somatic symptoms index” (D3; BEI = 1.09). Notably, the high-SA group exhibited the highest bridge symptom values, with “Depressed mood index” (D2; BEI = 2.17) emerging as the most prominent bridge node, followed by “Anxiety and panic” (A1; BEI = 1.08).

### 3.3. Network Robustness

Bootstrap results for all participants and three subgroups indicated that the 95% CIs for the edge weights were fairly narrow, demonstrating that the four networks have trustworthy edge estimates (Figure S1). The bootstrapped stability tests reflect a highly stable network across all participants and three subgroups (Figure S2). These findings indicate that the estimated node EIs exhibited excellent accuracy and stability. Additional results regarding network robustness are illustrated in Figure S3.

### 3.4. Network Comparison

After a two-by-two comparison of the low-, moderate-, and high-SA groups, there was no significant difference in global strength (*p* > 0.05) or network structure (*p* > 0.05).

### 3.5. Simulated Interventions for Different Groups

**Figure 4:**
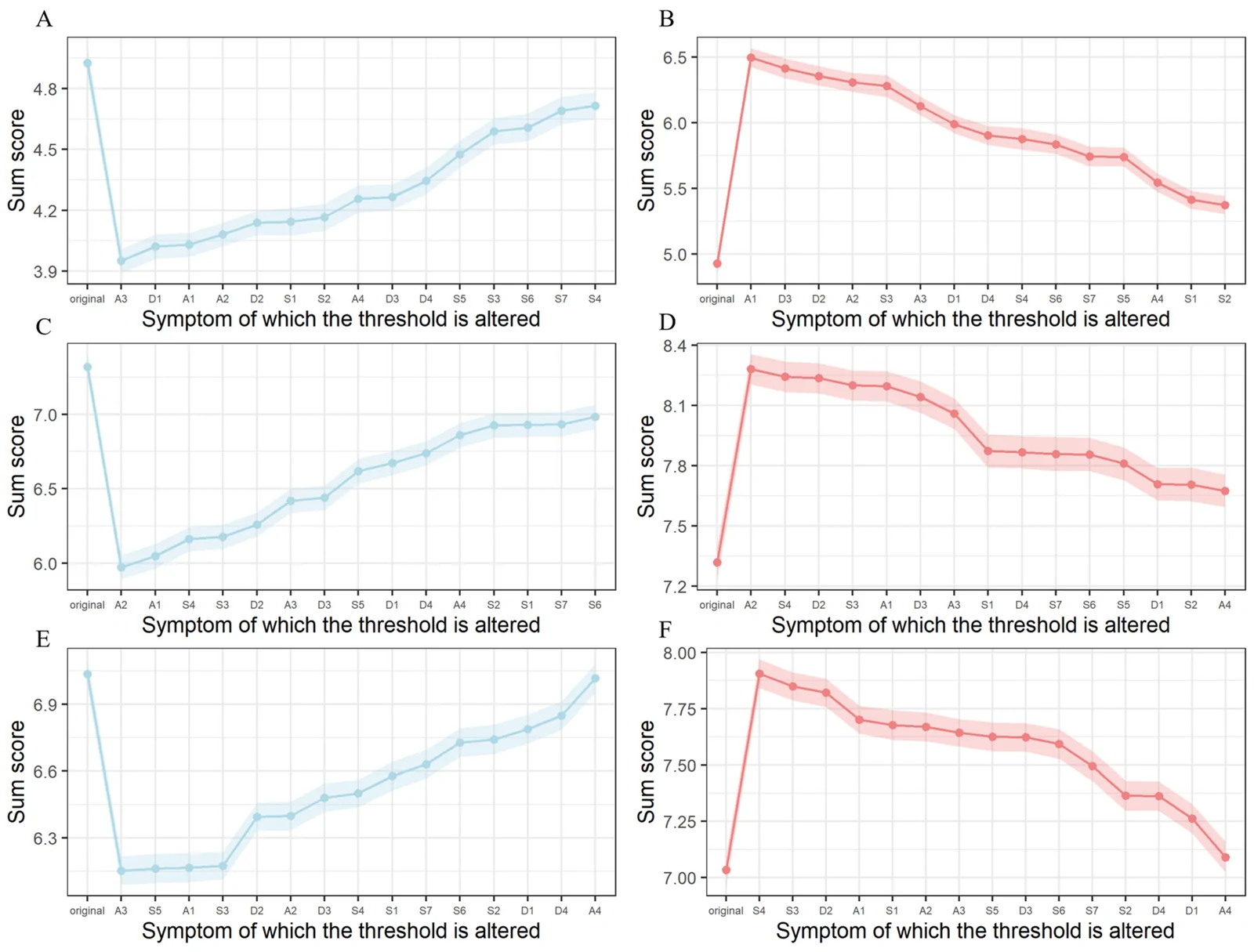
Alteration in total internalizing problems scores across three groups for mitigating and exacerbating interventions. (**A**) Simulated mitigation intervention in the low-SA group; (**B**) simulated exacerbation intervention in the low-SA group; (**C**) simulated mitigation intervention in the moderate-SA group; (**D**) simulated exacerbation intervention in the moderate-SA group; (**E**) simulated mitigation intervention in the high-SA group; (**F**) simulated exacerbation intervention in the high-SA group.

Based on the Ising model networks (Figure S4), separate alleviating and aggravating intervention simulations were conducted for each SA group. Results about robustness analysis in Ising networks were depicted in Figures S5–S7. The CS-C values for EI centrality were 0.36, 0.75, and 0.52 for the low-, moderate-, and high-SA groups, reflecting good stability of centrality estimates. As shown in Figure 4, the optimum intervention strategy for addressing overall internalizing problems networks differed across SA groups. Network comparison tests revealed significant differences in network structure between the low-SA and high-SA groups (*p* = 0.02) and between the moderate-SA and high-SA groups (*p* = 0.01). Specific differences in edge weights are presented in Table S2. The original sum scores were 7.03, 7.32, and 4.93 for the high-, moderate-, and low-SA groups, respectively.

With regard to alleviating interventions, reducing scores on each anxiety, depression, and sleep node led to varying degrees of reduction in overall internalizing problems. Specifically, reducing A3 resulted in the largest reduction in the low-SA group (Δtotal score = 0.98), while decreasing A2 had the largest influence in the moderate-SA group (Δtotal score = 1.34), and lowering A3 caused the most significant reduction in the high-SA group (Δtotal score = 0.88). These results indicate that reducing specific anxiety, depression, and sleep nodes can significantly influence overall internalizing problems across subgroups.

Considering the aggravating intervention, elevating A1 led to the highest increase in overall internalizing problems (Δtotal score = 1.57) in the low-SA group; in the moderate-SA group, increasing A2 had the largest influence (Δtotal score = 0.96); increasing S4 resulted in the biggest elevation (Δtotal score = 0.87) in the high-SA group, given that the simulated intervention effects carry the sampling variability of the estimated edge weights (Lunansky et al., 2022). Moreover, the differences in sum scores between the top-ranked and second-ranked candidate targets were at times modest. Consequently, these rankings should be interpreted as initial hypotheses for future prospective and experimental research rather than definitive statements about the superiority of one target over another.

By combining NAM with NIRA, this study identified specific key nodes associated with internalizing problems (Table S3). This finding is consistent with research showing that intervention response captures the network-wide influence of nodes more comprehensively than EI centrality indices (Machado et al., 2025). However, the NIRA simulations were based on cross-sectional Ising models and therefore cannot establish causality. These conclusions undoubtedly need to be treated with caution and require ongoing testing.

## 4. Discussion

In this study, participants were divided into three subgroups: low-, moderate-, and high-SA groups. We compared internalizing problems networks across three groups using a person-centered method, NAM and NIRA analysis, highlighting several key findings.

In the low-SA group, the strongest network connection occurred between the “Well-being index” (D1) and the “Optimism index” (D4). The finding indicates that individuals in the early stages of SA may seek comfort through digital media to cope with reduced well-being and increased pessimism. Prior studies have suggested that higher levels of hope in adolescents and greater optimism in college students are linked to lower levels of smartphone or internet addiction (Guo et al., 2020; Qiu et al., 2022). Research shows that positive psychological traits, including hope, optimism, and resilience, help protect against SA (Ji et al., 2025; Toma et al., 2022). SA can consume personal energy and increase stress, particularly when smartphones are used for hedonic purposes, such as gaming, entertainment or video streaming, which may in turn reduce overall well-being (Moqbel et al., 2023). Non-social smartphone use may serve as an avoidance coping strategy, more strongly predicting anxiety, depression, and problematic use than social-oriented use (Elhai et al., 2017). SA can further undermine well-being by intensifying social anxiety, weakening offline social connections, and increasing feelings of loneliness (Jing et al., 2025; P. Su & He, 2024). Individuals in the low-SA group may already face challenges in maintaining offline relationships, which could increase feelings of loneliness. This limited social support may contribute to reduced hope and positive expectations for the future, helping to explain the strong connection between lower well-being and pessimism in the low-SA group. While the strongest correlation was found between “Sleep duration” (S6) and “Sleep efficiency” (S7) in the moderate- and high-SA groups, these findings align with Cain and Gradisar’s (2010) three-pathway model, which explains how electronic media use disrupts sleep through interrelated mechanisms: (1) increased psychophysiological arousal due to stimulating content and interactive features, impeding sleep initiation; (2) time displacement, wherein media consumption encroaches upon allocated sleep time, resulting in delayed bedtimes and reduced sleep efficiency; and (3) light exposure, with short-wavelength blue light suppressing melatonin and upsetting circadian rhythms, thereby postponing sleep onset. A meta-analysis has linked SA to lower sleep efficiency and shorter sleep duration (Li et al., 2020). In conclusion, shortened sleep duration and reduced sleep efficiency form a strong association, suggesting that higher levels of SA may constitute a significant risk factor for sleep disorders.

Across both low- and moderate-SA groups, “Anxiety and panic” (A1) emerged as the most central node within the internalizing problems networks, highlighting its crucial role in sustaining these networks at lower levels of SA. These findings are supported by the compensatory internet use theory (CIUT), which suggests that people use online media to mitigate negative emotions (Kardefelt-Winther, 2014). The “Anxiety and panic” dimension includes symptoms of nervousness, unexplained fear, and being easily upset or panicky. Prior cross-sectional network studies have identified uncontrollable worry as the core node in anxiety–depression–sleep network among college students (Luo et al., 2025) and medical students in the anxiety–depression network (Chen et al., 2023), which aligns with the above findings. Furthermore, cross-lagged network analyses have found that PSU and anxiety symptoms interacted reciprocally, forming a vicious cycle of symptoms over time (Shen et al., 2024; Y. Wang et al., 2025). Fear-of-missing-out may constitute a key anxiety mechanism underlying SA, as habitual notification checking leads to a “reassurance-seeking” pathway that ultimately intensifies fear-of-missing-out (Billieux et al., 2015; Ratan et al., 2021). Overall, these results suggest that there may be a self-perpetuating cycle between anxiety symptoms and lower levels of SA. While in the high-SA group, “Sleep disturbances” (S3) became the most significant node, reflecting the severity of an individual’s inability to achieve restful, uninterrupted sleep due to various factors such as nightmares and breathing difficulties. Previous research has demonstrated that “Sleep disturbances” serves as a key bridge node linking problematic smartphone use with poor sleep quality (Zhou et al., 2025) and has been identified as the most prominent bridge symptom connecting PSU with depression, anxiety, stress, and sleep problems (Wu et al., 2026). These findings suggest that excessive smartphone use induces physiological and cognitive hyperarousal, making it difficult for individuals to relax and fall asleep (Nikolic et al., 2023). Moreover, blue light emitted from smartphone screens suppresses melatonin secretion and disrupts circadian rhythms, thereby contributing to sleep disruption (Tosini et al., 2016). The present results further support the internalization of conflicts model (Kales et al., 1976), which posits that persistent sleep disturbances hinder the processing and resolution of psychological conflicts, thereby impairing psychological regulation and exacerbating internalizing problems such as depression and anxiety (Baglioni et al., 2010). Consequently, in the high-SA group, “Sleep disturbances” (S3) functioned as a core node that sustains the internalizing problems network.

Bridge symptoms showed both shared and distinct patterns across the three SA subgroups. In the low-SA group, the “Somatic symptoms index” (D3) emerged as the bridge symptom with the highest BEI. This index primarily reflects tachycardia and fatigue. Prior research has shown that people with SA are more susceptible to neck and shoulder pain, which may progress into musculoskeletal disorders over time (Alotaibi et al., 2022). PSU is closely related to the symptoms of De Quervain’s tenosynovitis, with frequent thumb movements and improper grip posture being important contributing factors (Benites-Zapata et al., 2021). Moreover, prolonged improper smartphone-use postures may exacerbate multisite pain, thereby increasing perceived fatigue (Mustafaoglu et al., 2021). These results emphasize the significance of somatic symptoms in the preliminary phase of SA. In the moderate- and high-SA groups, the “Depressed mood index” (D2) was the primary bridge symptom connecting anxiety, depression, and sleep disorders. This dimension includes feeling depressed, crying spells, and suicidal rumination, commonly observed in individuals who use smartphones excessively to cope with emotional dysregulation (Ng et al., 2020). Depression is a core psychopathological feature comorbid with behavioral addictions (Zhao et al., 2023) and acts as a critical bridge connecting sleep disorders and mental health issues (Yu et al., 2024). Multiple studies have demonstrated that self-harm and suicidal ideation serve as critical bridge symptoms linking internalizing problems (Luo et al., 2025; Sun et al., 2024). Based on the interaction of person-affect-cognition-execution (I-PACE) model, when confronting stressful situations or negative affect, individuals may adopt impulsive emotion-regulation strategies, using smartphones to escape aversive cues (Brand et al., 2019; Hormes et al., 2014). While this provides short-term relief and social reassurance, it reinforces addiction cues, reduces sensitivity to natural rewards, and progressively impairs inhibitory control (Brand et al., 2016; Wegmann et al., 2018). Over time, excessive smartphone use becomes an automated, habitual response to alleviate depressive mood (Brand et al., 2019). Accordingly, the “Depressed mood index” becomes a central bridge symptom connecting the internalizing problems networks of moderate and high SA. Importantly, the above results support that SA arises from the interaction between two behavioral control systems. In the early or low-risk stages of SA, behavior is largely goal-directed and regulated by general inhibitory control. Smartphone use may function as a distraction or temporary relief from physical discomfort. However, as SA progresses to moderate and high levels, behavior becomes increasingly driven by immediate gratification and automatic processing, with reduced sensitivity to negative long-term consequences (Perales et al., 2020).

The NIRA analysis demonstrated that reductions in specific anxiety nodes were significantly associated with decreases in overall internalizing problems severity across all three SA groups. Specifically, “Gastrointestinal/Muscular sensations” (A3) exerted the strongest preventive impact in the low-SA group, “Vestibular sensations” (A2) in the moderate-SA group, and “Gastrointestinal/Muscular sensations” (A3) in the high-SA group. Conversely, “Anxiety and panic” (A1) exerted the strongest aggravating impact in the low-SA group, “Vestibular sensations” (A2) in the moderate-SA group, and “Use of sleep medication” (S4) in the high-SA group. Together, these findings underscore the important roles of specific anxiety and sleep nodes in both preventing and exacerbating overall internalizing problems among different groups.

Individuals with high SA exhibit more severe depressive symptoms and face a higher risk of disrupted melatonin secretion rhythms than low- and moderate-SA individuals (Srinivasan et al., 2009); they may be more inclined to seek pharmacological solutions to address reduced sleep duration and poor sleep efficiency (Nutt et al., 2008). So, “Use of sleep medication” (S4) became a crucial preventive target and had an influence on overall internalizing problems severity. Evening physical activities have been shown to reduce smartphone-related bedtime delay, with higher exercise frequency and longer duration being related to lower SA and anxiety levels (Y. Su et al., 2024). In clinical practice, health-promoting lifestyle is considered a widely applicable non-pharmacological approach for improving sleep quality (Zhang et al., 2023). Furthermore, specific anxiety nodes emerged as key intervention targets for reducing internalizing problems across SA severity levels. “Vestibular sensations” (A2), which reflect symptoms such as tremors, faintness, and paresthesias, and “Gastrointestinal/Muscular sensations” (A3), characterized by body aches and easy fatigability, were identified as optimal alleviating targets. Individuals with stronger, optimistic health mindsets tend to show greater willingness to engage in health-promoting behaviors, which may further support adaptive lifestyle changes and enhance health and well-being (Bunda & Busseri, 2019). Lifestyle patterns emphasizing stress management, psychological growth, and interpersonal relations are negatively correlated with anxiety (Kelly et al., 2020). Importantly, prior randomized clinical trials among breast cancer patients have shown that smartphone-based psychological interventions may offer a promising approach for reducing anxiety (Akechi et al., 2023). These results underscore the dual features of smartphones, with their effects largely dependent on usage patterns and contextual factors, thereby highlighting the importance of mindful smartphone use. Although the present results emphasize the essential roles of specific anxiety and sleep nodes in interventions, severity-specific intervention strategies tailored to different SA groups are warranted to effectively prevent and reduce overall internalizing problems.

## 5. Practical Implications

One of the main contributions of this study lies in providing severity-sensitive, symptom-level insights that may help inform tailored intervention strategies for individuals at different levels of SA severity. In the low- and moderate-SA groups, “Gastrointestinal/Muscular sensations” emerged as optimal alleviating targets. This finding suggests that interventions addressing anticipatory anxiety and physiological hyperarousal may be particularly beneficial at these stages. Cognitive–behavioral therapy (CBT) approaches appear well-suited, as they have demonstrated effectiveness in enhancing emotion regulation skills and reducing anxiety symptoms associated with maladaptive technology use (Hofmann et al., 2012). CBT-based interventions could incorporate components such as cognitive restructuring of catastrophic beliefs about disconnection, interoceptive exposure to reduce fear of physiological sensations, and skills training for managing anxiety during high-risk situations such as nighttime smartphone use.

In the high-SA group, however, “Sleep disturbances” became the most central node, and “Use of sleep medication” was identified as a potential aggravating target. This suggests that for individuals with severe SA, addressing sleep problems may be the initial therapeutic priority. Structured, sleep-focused interventions such as cognitive–behavioral therapy for insomnia (CBT-I), which includes stimulus control and sleep restriction, may be effective (Morin et al., 2006). Integrating psychoeducation on the effects of blue-light exposure and circadian rhythm disruption with behavioral sleep strategies may also strengthen educators’ ability to support individuals in establishing healthier routines (Cain & Gradisar, 2010). It is noteworthy that the majority of existing network analytic studies on SA and internalizing problems have focused on adolescent and university student populations (Wu et al., 2026; Zhou et al., 2025). Although the digital habits, developmental stages, and daily routines of these populations differ considerably from those of adults, similar results were observed, suggesting that the present study extends the applicability of previous findings beyond adolescent and university student samples.

## 6. Limitations and Future Directions

However, the present research has several limitations. Firstly, although NIRA has exhibited heightened clinical value relative to traditional methods that employ average scores of internalizing problems to ascertain intervention objectives (Machado et al., 2025), it cannot establish causality. Future longitudinal and experimental designs are necessary to validate the potential intervention targets identified in this study. Furthermore, considering that previous research has investigated gender discrepancies in experiencing anxiety, depression and sleep disorders, with females generally reporting higher levels of internalizing symptoms (Altemus et al., 2014), future research can examine network differences in internalizing problems among different levels of SA from a gender perspective. Emerging evidence suggests that the relationships among these variables may be both bidirectional and directional (Chellappa & Aeschbach, 2022; Karaş et al., 2023), highlighting a promising avenue for future research. Accordingly, longitudinal studies that integrate network analysis could be particularly valuable for examining these bidirectional and directed associations over time.

## 7. Conclusions

Our findings reveal several novel insights. Across three SA subgroups, “Anxiety and panic” emerged as the primary central node in low- and moderate-SA groups, whereas “Sleep disturbances” was the most central node in the high-SA group. In addition, the “Depressed mood index” served as a key bridge symptom, particularly among individuals with higher levels of SA, whereas the “Somatic symptoms index” showed the highest BEI in the low-SA group, indicating their pivotal roles in maintaining internalizing problems comorbidity across SA severity. NIRA analyses demonstrated that optimal symptom targets varied by SA severity. Specific anxiety nodes exerted the greatest influence in preventing and aggravating overall internalizing problems in the low- and moderate-SA groups, whereas specific sleep nodes, particularly “Daytime dysfunction” and “Use of sleep medication”, were influential in the high-SA group. Overall, the findings emphasize the significance of symptom-focused and severity-sensitive interventions, providing new insights for addressing internalizing problems across SA severity.

## Figures and Tables

**Table 1:** Demographic variables characteristics.

	*M*/*N*	*SD*/*%*
Age	28.87	10.17
Gender		
Female	644	45.64
Male	767	54.36
Subjective socioeconomic status		
Lowest level	118	8.36
Lower-middle level	415	29.41
Middle level	545	38.63
Upper-middle level	274	19.42
Highest level	59	4.18
Household registration		
Urban (Beijing)	554	39.26
Rural(Beijing)	250	17.72
Urban (other places)	424	30.05
Rural (other places)	183	12.97

**Table 2:** Fitting index of LPA based on SA.

Class	AIC	BIC	aBIC	Entropy	LMR (*p*)	BLRT (*p*)	Class Probability (%)
1	98,706.15	98,937.24	98,797.46	-	-	-	-
2	92,174.24	92,526.13	92,313.30	0.88	<0.001	<0.001	45.64/54.36
**3**	**89,223.04**	**89,695.73**	**89,409.83**	**0.92**	**<0.001**	**<0.001**	**19.63/21.26/59.11**
4	88,343.75	88,937.23	88,578.27	0.88	0.117	<0.001	8.29/15.59/29.70/46.42
5	87,553.72	88,268.00	87,835.98	0.90	0.395	<0.001	1.35/9.97/11.60/34.66/42.42

Note: Bold-faced class indicates the best-fitting model.

**Table 3:** Average latent class probabilities for most likely latent class membership.

Class	Class Counts	Class Probability (%)	Average Latent Class Probabilities		
			Class1	Class2	Class3
Class1	277	19.63	0.96	0.04	0.00
Class2	834	59.11	0.01	0.97	0.02
Class3	300	21.26	0.00	0.05	0.95

**Table 4:** Descriptive item statistics and differences among three SA groups.

Variables	Abb	Low SA Group (*n* = 277)		Moderate SA Group (*n* = 834)		High SA Group (*n* = 300)		*F*/*χ*^2^	ηp^2^	Pairwise Comparison
		*M*	*SD*	*M*	*SD*	*M*	*SD*			
Age		31.12	11.69	28.47	10.26	27.90	7.90	8.89 ***	0.01	
Gender (female%/male%)		23.91/16.04		58.54/59.58		17.55/24.38		18.82 _b_ ***	0.01	
Smartphone addiction		45.40	9.25	69.05	6.45	91.38	8.53	2679.25 ***	0.79	3 > 2 > 1
Withdrawal behavior	SA1	14.15	3.99	21.97	3.10	29.18	3.29	1467.12 ***	0.68	
Salience behavior	SA2	6.51	2.20	9.64	1.96	12.29	1.94	598.89 ***	0.46	
Social comfort	SA3	5.61	1.88	8.95	1.96	12.08	1.99	794.20 ***	0.53	
Negative effects	SA4	8.54	2.70	12.51	2.51	16.77	2.17	793.41 ***	0.53	
Use of application	SA5	7.04	2.43	10.09	1.91	12.97	1.57	659.15 ***	0.48	
Application renewal	SA6	3.56	1.40	5.89	1.68	8.11	1.60	574.10 ***	0.45	
Anxiety symptoms		35.58	8.92	45.19	8.89	52.47	9.05	259.07 ***	0.27	3 > 2 > 1
Anxiety and panic	A1	11.16	3.68	15.63	4.09	19.74	4.77	304.44 ***	0.30	
Vestibular sensations	A2	5.87	2.43	8.58	2.97	11.11	3.52	219.64 ***	0.24	
Gastrointestinal sensations	A3	6.70	2.25	9.16	2.54	11.55	3.03	250.56 ***	0.26	
Somatic control	A4	11.85	3.32	11.81	2.64	10.07	3.08	43.45 ***	0.06	
Depression symptoms		22.87	6.58	27.29	5.13	27.99	3.92	88.31 ***	0.11	3 > 2 > 1
Well-being index	D1	10.73	3.45	11.70	2.77	10.28	3.03	30.04 ***	0.04	
Depressed mood index	D2	4.75	1.83	6.53	2.05	8.07	2.32	185.68 ***	0.21	
Somatic symptoms index	D3	3.21	1.34	4.44	1.58	5.45	1.64	150.51 ***	0.18	
Optimism index	D4	4.18	1.65	4.62	1.38	4.20	1.33	15.27 ***	0.02	
Sleep symptoms		4.73	3.19	7.31	3.60	8.49	3.98	83.48 ***	0.11	3 > 2 > 1
Subjective sleep quality	S1	0.81	0.71	1.10	0.82	1.25	0.93	21.26 ***	0.03	
Sleep latency	S2	0.82	0.75	1.14	0.77	1.20	0.76	22.25 ***	0.03	
Sleep disturbances	S3	1.01	0.67	1.53	0.73	1.83	0.87	87.47 ***	0.11	
Used sleep medication	S4	0.33	0.70	0.88	0.97	1.22	1.12	63.37 ***	0.08	
Daytime dysfunction	S5	1.04	0.85	1.56	0.88	1.79	0.90	56.49 ***	0.07	
Sleep duration	S6	0.35	0.67	0.54	0.84	0.53	0.88	5.70 **	0.01	
Sleep efficiency	S7	0.37	0.80	0.56	0.96	0.68	1.06	7.67 ***	0.01	

Note: *M*: mean; *SD*: standard deviation; Abb: abbreviation; gender: female = 0, male = 1; Chi-square values: indicated by the subscript b, represent differences in the distribution of specific demographic categories between groups. ** *p* < 0.01, *** *p* < 0.001.

## Data Availability

The data are not publicly available due to privacy or ethical restrictions, but they will be available from the corresponding author upon reasonable request.

## Supplementary Materials

The following supporting information can be downloaded at: https://www.mdpi.com/article/10.3390/bs16081318/s1, Figure S1: Accuracy of edges weights in Gaussian Graphical Model (GGM) networks; Figure S2: Stability of edges estimation in GGM networks; Figure S3: Bootstrapped difference tests in GGM networks; Figure S4: Ising networks estimated for different groups; Figure S5: Accuracy of edges weights in ising networks; Figure S6: Stability of edges estimation in ising networks; Figure S7: Bootstrapped difference tests in ising networks; Table S1: The bridge expected influence (BEI) values in GGM networks among all participants and three subgroups; Table S2: The Edge Invariance Test; Table S3: Comparison of key nodes between NIRA Analysis and Network Analysis.

## Abbreviations

The following abbreviations are used in this manuscript:

SA	Smartphone addiction
PSU	Problematic smartphone use
LPA	Latent profile analysis
NAM	Network analysis method
NIRA	NodeIdentifyR Algorithm
AIC	Akaike information criterion
BIC	Bayesian information criterion
aBIC	Adjusted Bayesian information criterion
BLRT	Bootstrap likelihood ratio test
LMR	Lo–Mendell–Rubin likelihood ratio
LASSO	Least absolute shrinkage and selection operator
EBIC	Extended Bayesian information criterion
EI	Expected influence
BEI	Bridge expected influence
CS-C	Correlation stability-coefficient
