# Supplementary material for: Differences in Internalizing Problems Across Risk Groups of Smartphone Addiction: Simulation-Based Network Analysis of Intervention Targets
Source: Behav Sci (Basel). 2026 Aug 3;16(8):1318. doi: 10.3390/bs16081318 (PMC13509764; doi:10.3390/bs16081318)
Supplement: Supplementary file 1 [file behavsci-16-01318-s001.zip › behavsci-4402558-supplementary.pdf]

Supplementary Materials

Supplementary Figure S1. Accuracy of edges weights in Gaussian Graphical Model (GGM) networks.

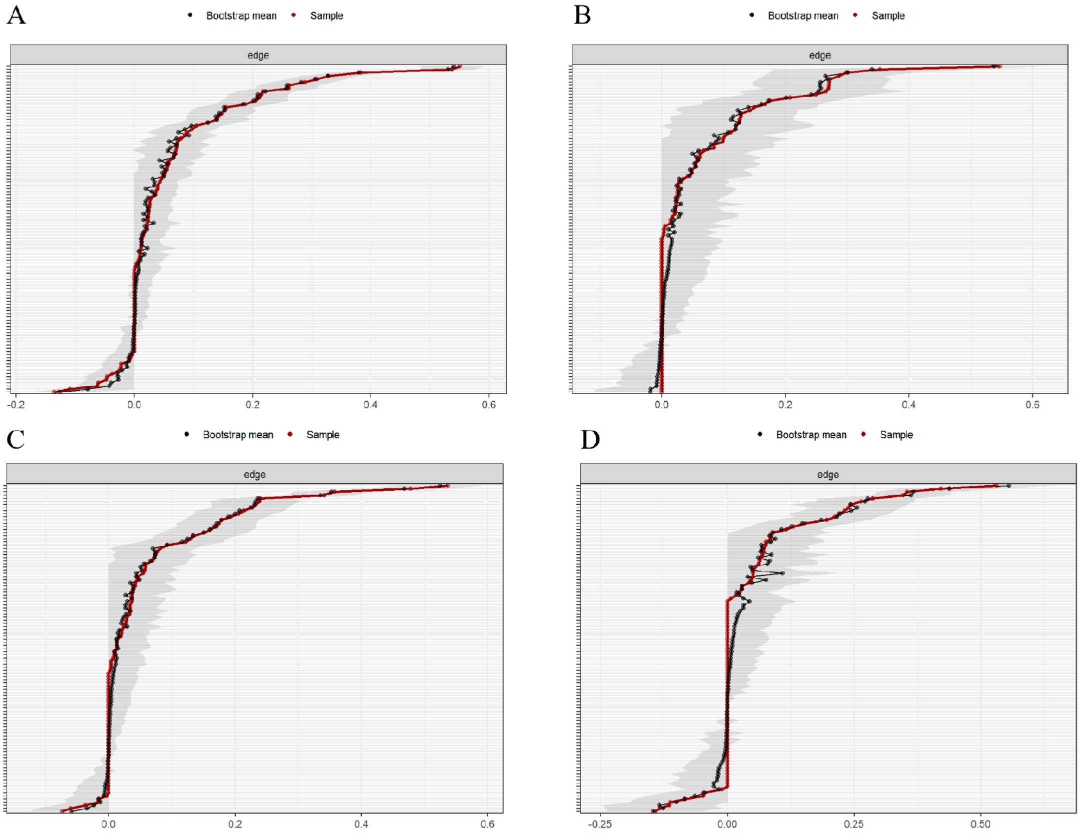

Note: The red line depicts the sample edge weights and the gray bar depicts the bootstrapped confidence interval.

Supplementary Figure S2. Stability of edges estimation in GGM networks.

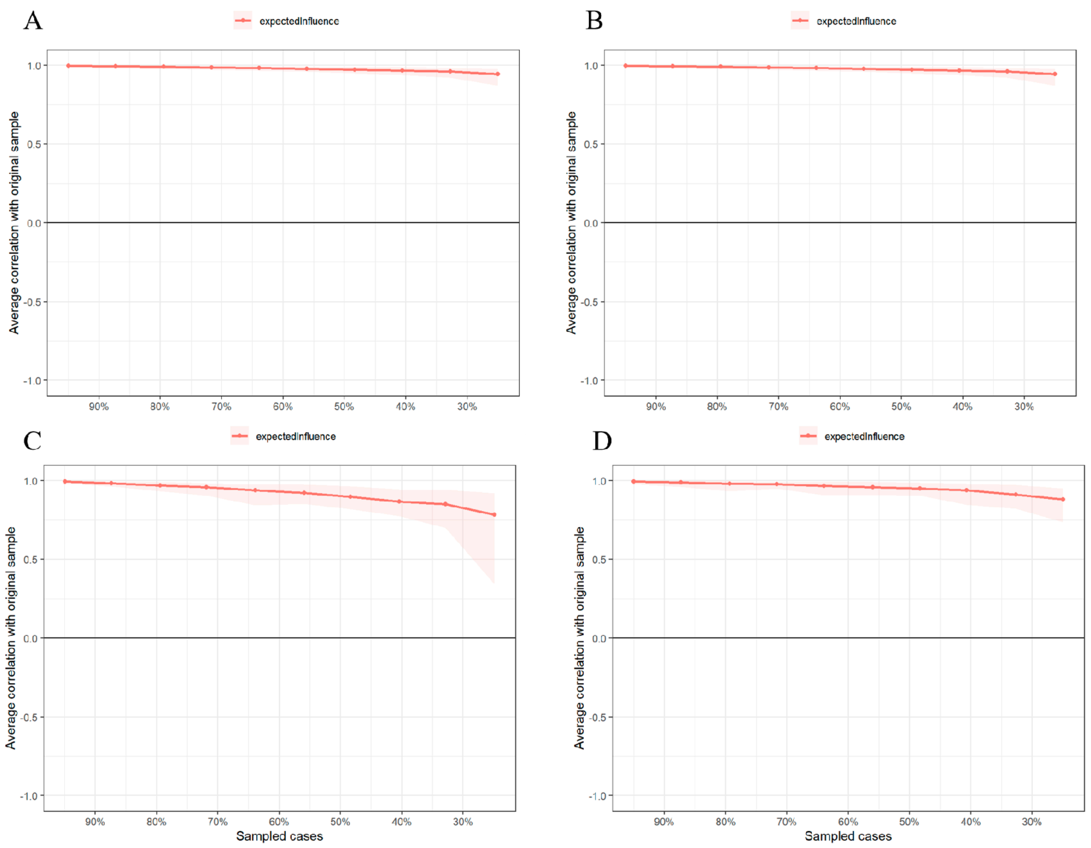

Note: The red bar represents the average correlation between node expected influences in the full sample and subsample with the red area depicting the 2.5th quantile to the 97.5th quantile.

Supplementary Figure S3. Bootstrapped difference tests in GGM networks.

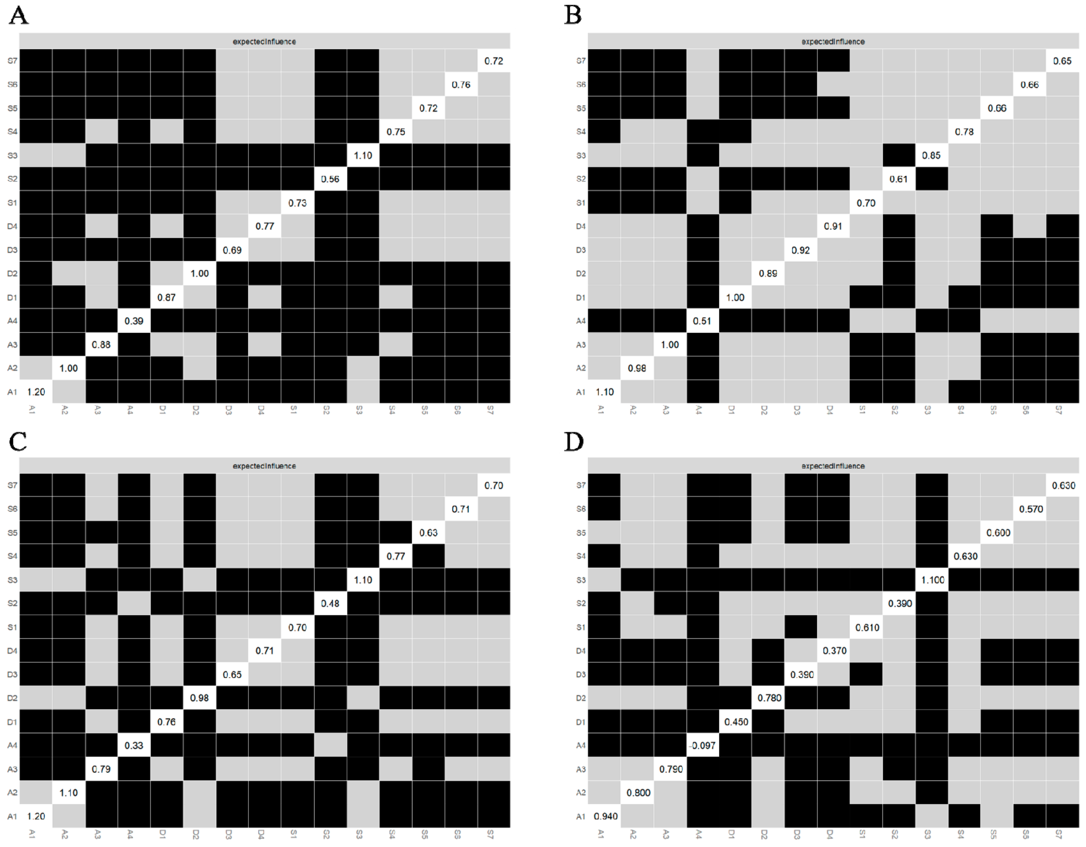

Note: Gray boxes indicate node expected influences that do not differ significantly from one another, while black boxes indicate node expected influences that do differ significantly. The numbers in the white boxes (i.e., diagonal line) represent the values of node expected influences.

Supplementary Figure S4. Ising networks estimated for different groups.

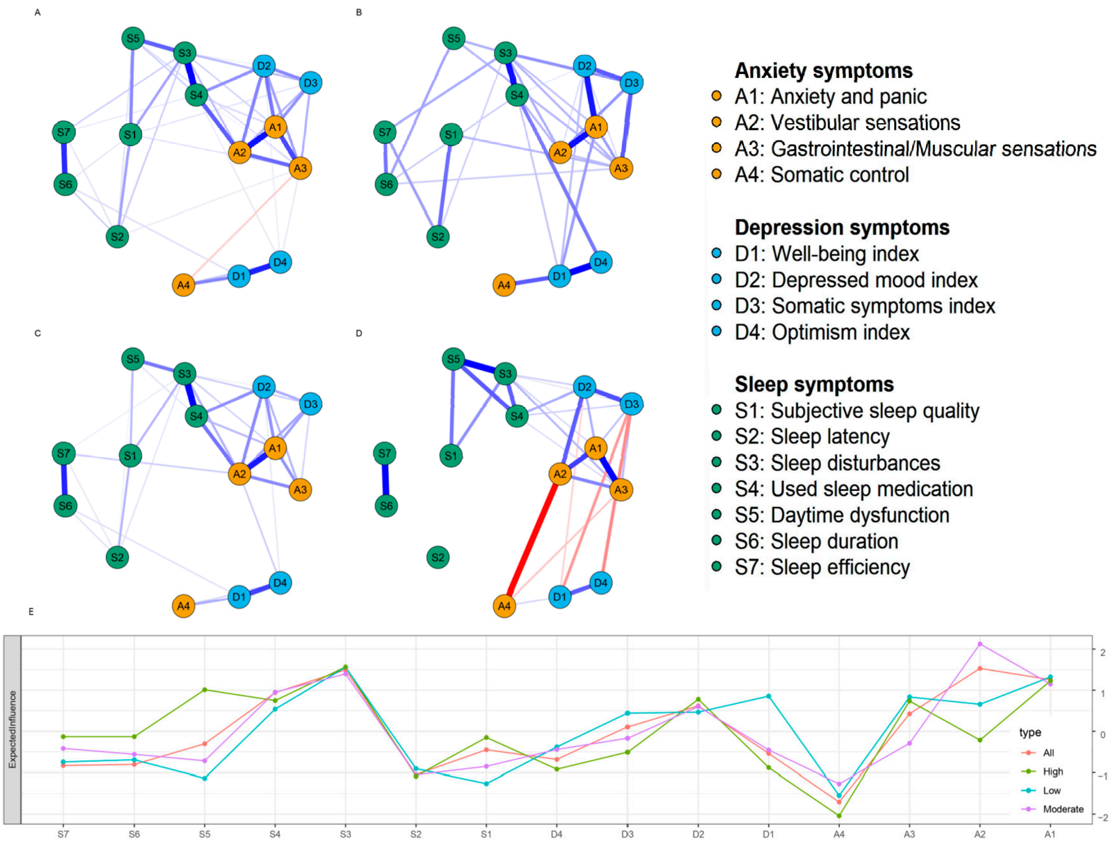

Note: Anxiety-depression-sleep networks of (A) all participants, (B) low SA group, (C) moderate SA group, and (D) high SA group and Z-values of node EI centrality in the (E): All represents entire sample; Low represents low SA group; Moderate represents moderate SA group; High represents high SA group. Nodes represent anxiety, depression, and sleep symptoms, and edges represent regularized partial correlations between nodes. The blue edges represent positive correlations, and the red edges represent negative correlations.

Supplementary Figure S5. Accuracy of edges weights in ising networks.

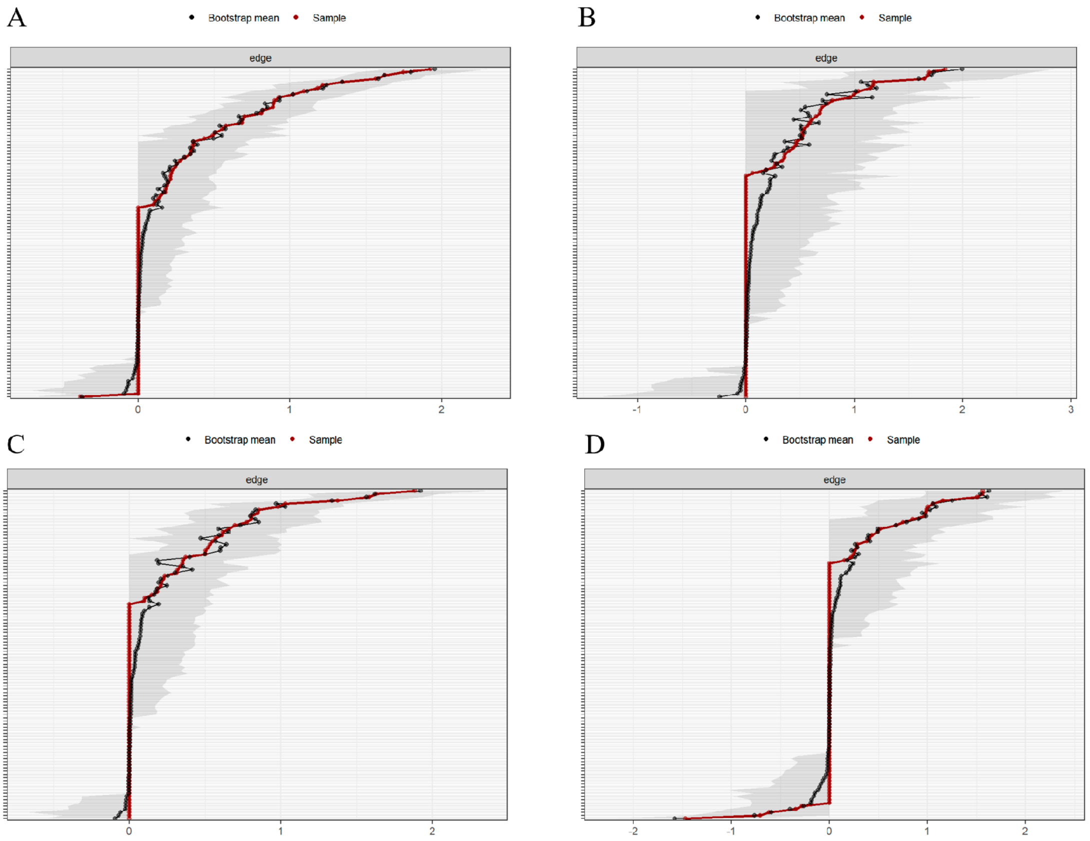

Note: The red line depicts the sample edge weights and the gray bar depicts the bootstrapped confidence interval.

Supplementary Figure S6. Stability of edges estimation in ising networks.

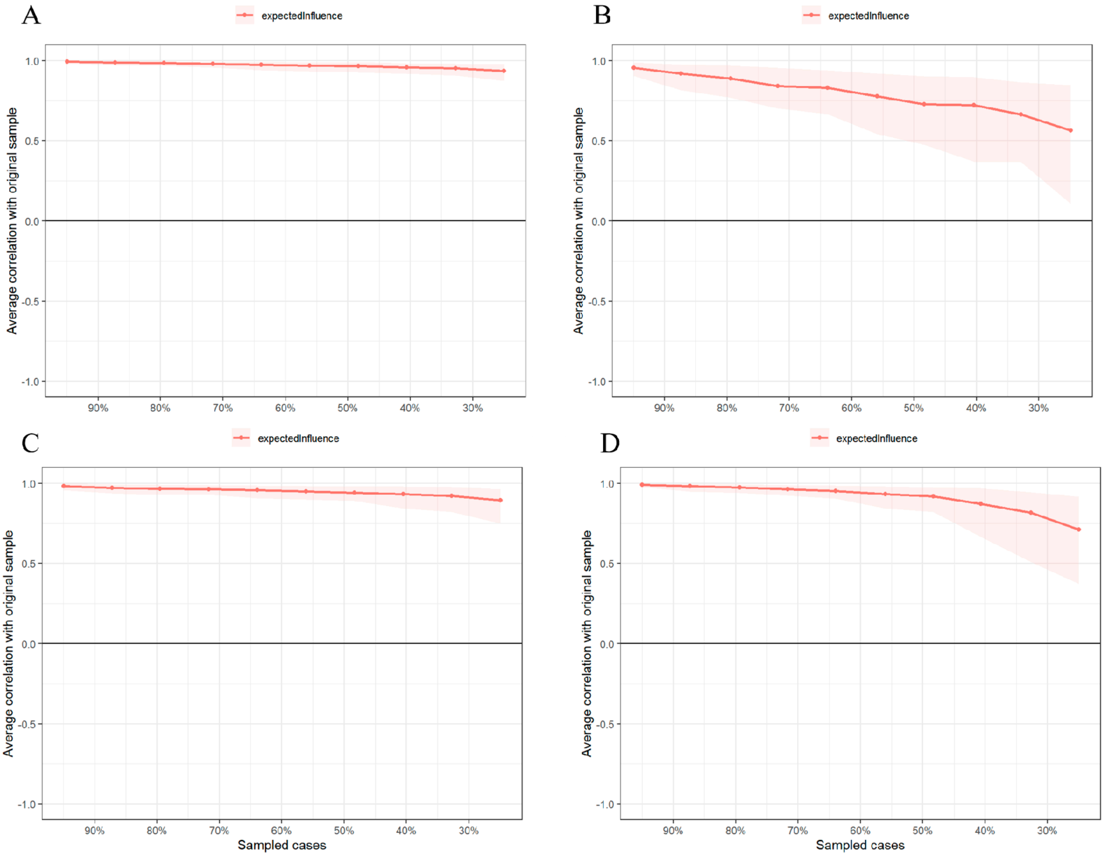

Note: The red bar represents the average correlation between node expected influences in the full sample and subsample with the red area depicting the 2.5th quantile to the 97.5th quantile.

Supplementary Figure S7. Bootstrapped difference tests in ising networks.

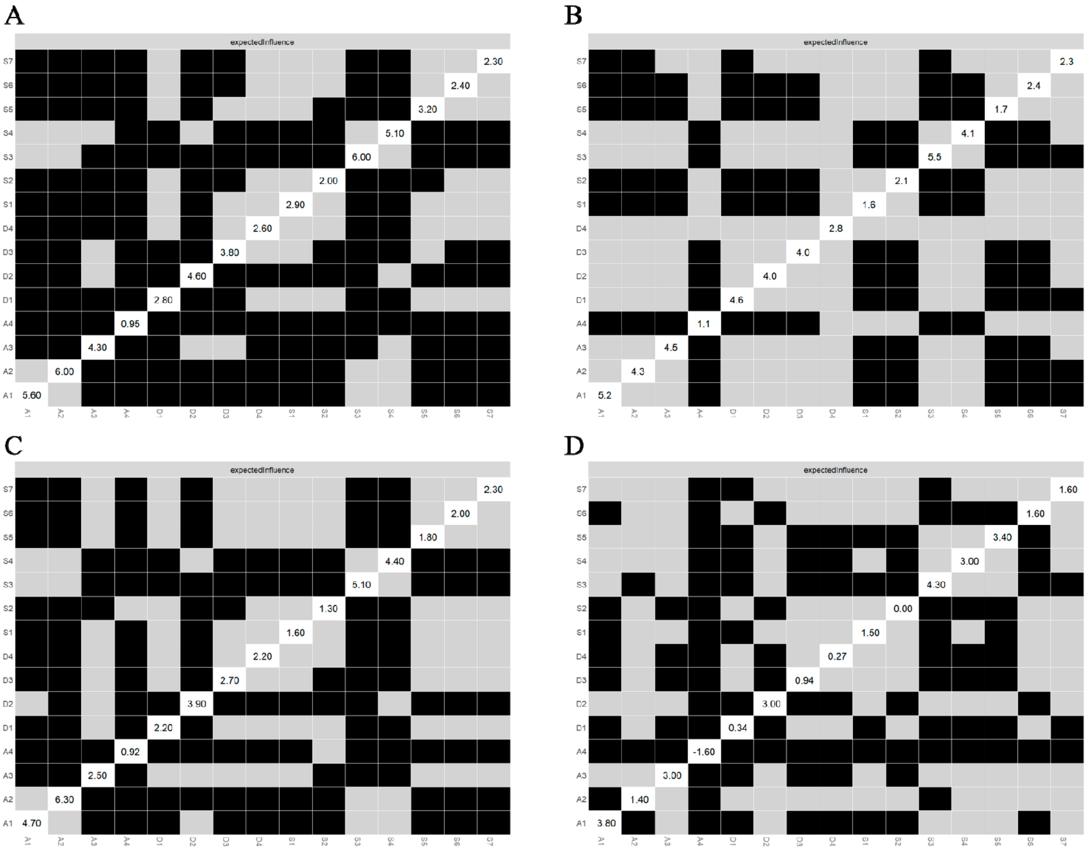

Note: Gray boxes indicate node expected influences that do not differ significantly from one another, while black boxes indicate node expected influences that do differ significantly. The numbers in the white boxes (i.e., diagonal line) represent the values of node expected influences.

Supplementary Table S1. The bridge expected influence (BEI) values in GGM networks among all participants and three subgroups.

All participants		Low SA group		Moderate SA group		High SA group	
Node	BEI	Node	BEI	Node	BEI	Node	BEI
D2	2.03	D3	1.50	D2	2.15	D2	2.17
A4	1.19	D2	1.00	A1	1.14	A1	1.08
D3	1.05	A3	0.95	D3	1.09	D3	0.93
A1	0.86	A4	0.85	A2	0.76	A2	0.87
A3	0.57	A1	0.79	A3	0.46	A3	0.43
A2	0.32	S4	0.66	A4	0.43	A4	0.36
D1	0.09	A2	0.47	S4	0.04	S3	0.16
S4	-0.12	D4	0.18	D1	-0.05	S4	0.03
D4	-0.17	D1	0.13	S3	-0.35	D1	-0.09
S3	-0.42	S3	-0.48	D4	-0.40	S2	-0.63
S5	-0.85	S5	-0.89	S5	-0.85	S7	-0.79
S2	-0.95	S7	-1.08	S6	-1.04	D4	-0.85
S7	-1.07	S1	-1.22	S7	-1.04	S1	-1.22
S6	-1.15	S6	-1.42	S2	-1.11	S5	-1.22
S1	-1.38	S2	-1.45	S1	-1.22	S6	-1.22

Supplementary Table S2. The Edge Invariance Test.

Low SA group and high SA group			Moderate SA group and high SA group		
Edges	*E*	*p*	Edges	*E*	*p*
A2 - A4	1.47	0.0099	A2 - A4	1.47	0.0099
A1 - D2	1.16	0.0396	A2 - S4	1.03	0.0099
S1 - S2	1.04	0.0396	S3 - S4	0.91	0.0297
S3 - S5	1.00	0.0297	S4 - S5	0.79	0.0198
D4 - S4	0.96	0.0099	D3 - D4	0.71	0.0297
D1 - D4	0.86	0.0396	D1 - D3	0.62	0.0297
S1 - S5	0.85	0.0099	A2 - S3	0.57	0.0297
D1 - D2	0.75	0.0099	D1 - D2	0.28	0.0099
D3 - D4	0.71	0.0099	D2 - D4	0.19	0.0198
A1 - D1	0.70	0.0099			
S2 - S7	0.69	0.0099			
S1 - S3	0.68	0.0099			
S5 - S6	0.65	0.0495			
D1 - D3	0.62	0.0099			
A2 - S3	0.59	0.0198			
A3 - S5	0.52	0.0099			
D1 - S4	0.39	0.0198			
A3 - S6	0.36	0.0396			
S4 - S6	0.36	0.0099			
S2 - S3	0.33	0.0099			

Note: *E* = absolute difference in edge weights between the two networks. All *p*-values are uncorrected for multiple comparisons.

Supplementary Table S3. Comparison of key nodes between NIRA analysis and network analysis.

Group	NIRA analysis				Network analysis	
	Optimal alleviating intervention node	Δ Total Score	Optimal aggravating intervention node	Δ Total Score	Highest EI node	EI Value
Low SA group	A3(Gastrointestinal /Muscular sensations)	-0.98	A1(Anxiety and panic)	1.57	A1(Anxiety and panic)	1.39
Moderate SA group	A2(Vestibular sensations)	-1.34	A2(Vestibular sensations)	0.96	A1(Anxiety and panic)	1.73
High SA group	A3(Gastrointestinal /Muscular sensations)	-0.88	S4(Used sleep medication)	0.87	S3(Sleep disturbances)	1.77

NIRA = NodeIdentifyR Algorithm; EI = expected influence. Δ Total Score evaluated the impact on overall internalizing problems symptoms, with higher scores indicating improvement.
